# Nanochannel Electroporation as a Platform for Living Cell Interrogation in Acute Myeloid Leukemia

**DOI:** 10.1002/advs.201500111

**Published:** 2015-07-16

**Authors:** Xi Zhao, Xiaomeng Huang, Xinmei Wang, Yun Wu, Ann‐Kathrin Eisfeld, Sebastian Schwind, Daniel Gallego‐Perez, Pouyan E. Boukany, Guido I. Marcucci, Ly James Lee

**Affiliations:** ^1^ Center for Affordable Nanoengineering of Polymeric Biomedical DevicesThe Ohio State UniversityColumbusOH43212USA; ^2^ William G. Lowrie, Department of Chemical and Biomolecular EngineeringThe Ohio State UniversityColumbusOH43210USA; ^3^ Department of Internal MedicineComprehensive Cancer CenterThe Ohio State UniversityColumbusOH43210USA

**Keywords:** nanochannel electroporation, living cell interrogation, RNA recognition, fluorescent probes

## Abstract

**A living cell interrogation platform based on nanochannel electroporation** is demonstrated with analysis of RNAs in single cells. This minimally invasive process is based on individual cells and allows both multi‐target analysis and stimulus‐response analysis by sequential deliveries. The unique platform possesses a great potential to the comprehensive and lysis‐free nucleic acid analysis on rare or hard‐to‐transfect cells.

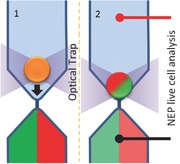

A comprehensive micro/nanofluidics platform for single‐cell analysis based on nanochannel electroporation (NEP) and molecular beacon (MB) is presented in this study. The platform can quantitatively analyze multiple RNA species in individual cells with minimal cell damage. Furthermore, it is capable of delivering nucleic acids into target cells and subsequently detecting their responses at RNA level, e.g., microRNA (miRNA). It is known that as the downstream targets of miR‐29b, DNMT3A/B can be downregulated by miR‐29b overexpression. To demonstrate the activity of delivered miR‐29b by NEP and the analytical function of the platform, the decreased expression of DNMT3A/B in acute myeloid leukemia (AML) cells was verified at single‐cell level by simultaneous detection of multiple genes in the same cell. The potential of such platform on intracellular pathway studies has also been explored by investigating the upregulation efficiencies of miR‐181a through different pathways in AML cells. The results showed that an indirect approach by C/EBPα‐p30 peptide expression would have a stronger effect than direct transfection of the miR‐181a gene. The platform has also shown its advantages over established technologies in the analysis of cells that are hard to transfect.

Stimulated by demand from biological and medical communities, the development of single‐cell analysis technologies has received broad attention in recent years.^1^ Unlike established methods for large cell populations, single‐cell analysis technologies generally focus on a small number of cells and can provide differentiation of biological activities at a resolution of intracellular level. In the case of limited cell population, such technologies are the only analytic option. Due to the small size of a single cell, lab‐on‐a‐chip devices with micro/nanoscale features are an ideal fit. The demand of single‐cell analysis has also been expanded from mere sorting‐and‐identification to multiple analyses and responsive studies for treatments in living cells. To address these demands, we have developed a new platform based on a novel single‐cell transfection tool—NEP.^2^ NEP functions as a delivery platform to individual cells. The major strengths of NEP are the minimal cellular disturbance and precise delivery dosage.^2^, ^3^ Optical tweezers are used for single‐cell manipulation on an NEP device, so the operation is typically based on an inverted microscope with a high numerical water/oil immersion objective lens, which provides a high spatial resolution down to 200 nm. These characteristics (both from NEP itself and its operation) have prepared NEP as an analytical tool for cellular contents, with the sample size down to a single cell and the resolution at the organelle level.

Specifically, single‐strand nucleic acids inside a cell, such as messenger RNAs (mRNAs) and micro RNAs (miRNAs), can be quantitatively detected by MBs delivered by NEP.^4^ Both mRNAs and miRNAs play critical roles in gene expression and regulation;^5^ therefore, their analyses at a single‐cell level can provide significant information for cell biology. MBs are synthetic linear nucleic acids with a signaling molecule (typically a fluorescent dye) conjugated to one end, and its corresponding quencher to the other. MBs have two‐end sequences that complement each other to form the stem, sandwiching a non‐self‐complementary sequence (loop) that recognizes its target. Without its target, the MB folds up as a hairpin structure and the quencher locates in proximity to the dye, allowing energy transfer that quenches the fluorescence. Upon encountering its target, the beacon unfolds and binds to the target, allowing the dye to emit fluorescence. Since the fluorescence intensity is proportional to the count of freed dye molecules, it is used in both qualitative and quantitative detections of DNA and RNA.^4^, ^6^

The delivery function of NEP and the analysis function of MBs can be combined to examine the genetic response of individual cells to foreign materials, which provides a living cell interrogation platform at the single‐cell level. To do so, NEP is first conducted to deliver a specified amount of molecules of interest into the target cells. After the delivered materials have induced cellular response at mRNA or miRNA level, a second NEP is conducted to deliver MB for the detection of such a response. Among many potential applications of NEP based probing and detection is the gene expression and regulation pathway study of rare cells obtained from biopsy, of which most existing analytical methods cannot be applied due to the very small sample size. Living cell interrogation is also impossible in most existing methods because cells need to be fixed or lysed for characterization. Such studies include the restoration or knockdown of an upstream gene and the detection of expression change for a downstream gene. This platform may also be utilized to establish intracellular pathways for cells that are resistant to conventional transfection methods. Here, we show two examples of NEP‐based living cell interrogation by delivering either oligonucleotides or plasmids carrying exogenous genes. The ability of analyzing multiple targets in a single living cell is also demonstrated.

In this work, we used two AML cell lines, Kasumi‐1 and KG1a, and two microRNAs, miR‐29b and miR‐181a, whose antileukemia potential have been shown,[[qv: 5b]],^7^ as study examples to demonstrate a comprehensive nucleic acid analysis strategy on the NEP living cell interrogation platform. Both Kasumi‐1 and KG1a have low expression of miR‐29b and miR‐181a. In addition, Kasumi‐1 has been shown to have overexpressed DNMT3A and DNMT3B,[[qv: 5b]],[[qv: 7b]] and KG1a does not have observable CEBPα level.^8^

A silicon template with the nanoridge dimensions of 5 μm (L) × 200 nm (W) × 200 nm (H) and a microridge cross section of 40 μm by 40 μm was fabricated by photolithography, as described previously.^9^ The nanoridge connects a pair of microridges at the bottom of their pointing tips. To produce the micro/nano/micro‐channel structure for NEP, the template was vapor primed by hexamethyldisilazane (HMDS) and coated with a 1:1 mixture of the base resin and curing agent from Sylgard 184 PDMS kit (Dow Corning). The template and mixture were then baked at 60 °C for 45 min before applying on the top a layer of 10:1 mixture of PDMS from the same kit. The total thickness of the PDMS on silicon template was 3 ± 0.5 mm. The PDMS block was de‐molded from the template after curing of the second PDMS layer, and its corners were removed by a blade. Two reservoirs were punctured to allow fluidic and electric access to each side of the microchannels. Dust and particles on the PDMS block were removed by an adhesive tape. A No.1 coverglass was first cleaned by sonication in isopropanol and deionized water, then dried by particle‐filtered airflow. The PDMS block and coverglass were treated by ozone (UVO cleaner Model 42, Jelight Company Inc.) for 5 min, followed by immediate bonding to form the NEP device. The sealing produced in this way is reversible, yet strong enough to resist 70% ethanol for a short period (i.e., 1 h) for sterilization. The device was then immersed in 70% ethanol for 10 min and placed under UV light in a biological safety cabinet for at least 20 min for disinfection. After UV exposure any residual ethanol was aspirated out and the device was kept in a sterilized container for further usage. **Figure** **1** demonstrated the fabrication process of NEP device with optical images of the fabricated PDMS block as well as a final functioning device.

**Figure 1:**
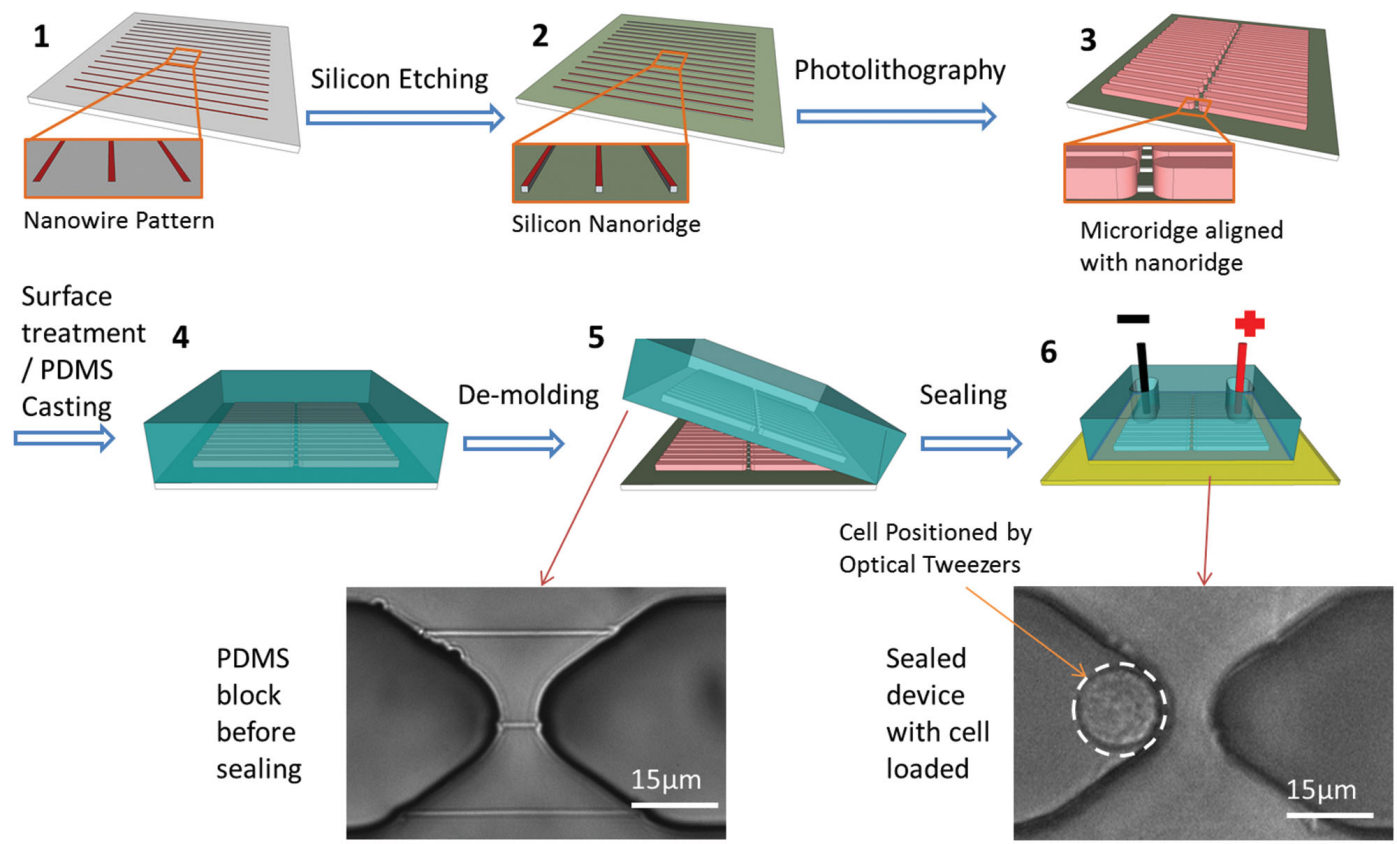
The fabrication and assembling process of NEP device (from step 1 through step 6). The solution reservoirs are omitted in step 6 for a clear view. Nanochannels are more visible on PDMS before sealing on coverglass due to higher refractive index of glass than air.

A prepared device was filled with a nucleic acid solution and a cell suspension on each side. The device was incubated to allow for the diffusion of miRNA or MBs into microchannels as well as to warm up the fluid in the cell side reservoir. The loaded device was mounted onto an inverted fluorescence microscope with optical tweezers to manipulate individual cells into individual microchannels against the tips of nanochannels.^2^ A pair of platinum wires (250 μm diameter) connected to an electroporation power supply (Gene Pulser Xcell, Bio‐Rad) was immersed vertically into the two reservoirs at a depth of 3 mm to act as electrodes. Two to five electric pulses between 220 and 250 V, each lasting 5 or 10 milliseconds, were delivered with a square wave protocol to conduct cell transfection. While electrolysis on the surface of Pt electrodes might change the local solution composition near electrodes in the reservoir, microchannels provided additional diffusion length. Therefore, the medium composition near cell (half‐way between two electrodes) would only experience minimal disturbance. When multiple nanochannel connections exist between two microchannels (see Figure 1), the electrophoresis is dominated by the path of the lowest resistance, therefore no adjustment on the pulsing condition is needed as long as the cell only touches one nanochannel or if other nanochannels are significantly longer than the touching nanochannel. Unlike conventional bulk electroporation, NEP transfects individual cells with minimal damage in tightly controlled microenvironments and can achieve high efficiency without viability loss. The transfection efficiency and cell viability were close to 100% at conditions used in this work, which is consistent with our previous findings on a variety of cells including the KG‐1a cell used here.^2^, ^3^, ^9^

MiRNAs are a family of short non‐coding RNAs that participate in RNA interference and regulate gene expression at the post‐transcription level by selectively binding to and silencing target mRNAs. Since complete complimentary binding is not required for silencing, one miRNA could have more than one target. For example, miR‐29b has been shown to be deregulated in AML and directly targets DNA methyltransferases, DNMT3A and 3B.[[qv: 5b]],[[qv: 7b]] Although the bulk population transfected with either miR‐29b synthetic mimic molecules or miR‐29b expression plasmid showed decreased level of DNMT3A and 3B, there is no evidence to show the direct correlation between miR‐29b and DNMT3A and 3B in a single cell.

In this study, MBs for DNMT3A and DNMT3B mRNAs were designed with different fluorescent molecules to detect both in the same cell. Notably, to demonstrate the simultaneous detection of multiple targets, DNMT3A and DNMT3B MBs were delivered by NEP into Kasumi‐1 cells (an AML cell line). The green and infrared fluorescence levels of individual cells were used to quantify the expression level of each mRNA, respectively. Cells positioned in the microchannel but away from the nanochannel received no MBs and served as a negative control. Typical fluorescence and phase contrast images of control and MB‐transfected Kasumi‐1 cells are shown in **Figure** **2**a,b. Both MBs were able to detect their corresponding mRNAs, with the fluorescence showing a subtle difference on the intracellular distribution of DNMT3A and 3B. Control cells showed little background fluorescence.

**Figure 2:**
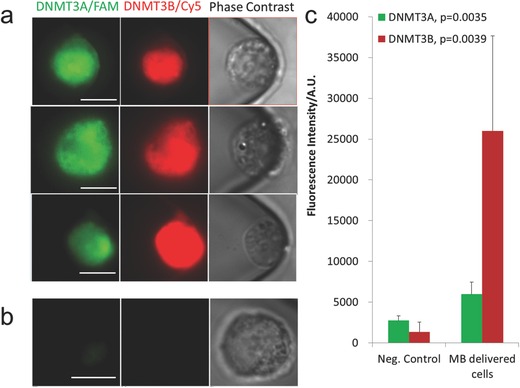
a) Fluorescence and phase contrast images of three individual wild‐type Kasumi‐1 cells transfected with DNMT3A/B MBs; b) those of a negative control cell; c) Average fluorescence signals from MB transfected Kasumi‐1 cell (*n* = 7) and negative control (*n* = 3). *P*‐values in legend are from one side *t*‐test assuming equal variance.

For quantitative comparison, the average fluorescence intensities in MB‐transfected and control Kasumi‐1 cells were recorded and measured by software as the basal expression level of DNMT3A/B, and compared in Figure 2c. Fluorescence signals from both MBs are statistically higher than those from negative controls, with a *P*‐value <0.01, indicating the successful detection by MBs. The elevated standard deviation in the transfected cell group reflects natural fluctuation of the expression of mRNAs. The mRNA number between individual Kasumi‐1 cells measured from MB fluorescence varies by 1.9 folds for DNMT3A and 4.3 folds for DNMT3B, further emphasizing the need of multi‐target analysis on single cells.

Next we delivered miR‐29b synthetic mimic molecules into Kasumi‐1 cells and detected the expression level change of DNMT3A and 3B with a second NEP of MBs 24 h later. The fluorescence signals from miR‐29b transfected cells were measured by the aforementioned method, and then compared with the signal level in untreated Kasumi‐1 cells in Figure S1 (Supporting Information). The delivered miR‐29b mimic significantly downregulated the mRNA level of DNMT3A and 3B, which is consistent with the established intracellular pathway.[[qv: 7b]] This experiment successfully demonstrated NEP/MB‐based mRNA analysis in single living cells.

For the NEP‐based multi‐target analysis demonstrated in this work, the number of targets that could be analyzed simultaneously in one cell is limited primarily by the potential cross‐talk between the fluorescence channels. The use of fluorescence probes with a narrow emission (i.e., quantum dots) paired with narrow band‐pass filter cubes or microscopic fluorescence spectrometer could lift such limitation and improve the multi‐targeting ability.[[qv: 6a]]

With the multi‐targeting function demonstrated, the single‐cell NEP analysis system has next been used to explore more complicated intracellular pathways. In this part of study, the regulation strength of mutated CCAAT/enhancer‐binding protein alpha (CEBPA) gene on miR‐181a expression in AML cells was explored and compared with miR‐181a plasmid reference.

The CEBPA gene encodes a transcription factor that plays vital function in hematopoiesis. It has two isoforms, p30 and p42.[[qv: 7c]] However, frequent CEBPA abnormalities in AML patients have been observed clinically. Specifically, point mutations were widely observed in AML patients,^10^ with 90% of them containing N‐terminal frame‐shift mutations.^11^ Such nonsense mutations would prevent correct translation of the full length C/EBPα peptide, but the expression of a truncated isoform (C/EBPα‐p30) is not affected. On the other hand, a C‐terminal mutation could disrupt the conservative sequence for dimerization and DNA binding, rendering both isoforms functionless.^12^ Interestingly, in most clinical cases, patients show both N‐terminal mutation and C‐terminal mutation, but on separate alleles. Such double mutations would result in the translation and function of only p30 isoform and no p42 peptides. Compared to the relative rare case of single mutation, these biallelic double CEBPA mutations have more favorable prognostic impact,^13^ yet the mechanism behind such favorable prognosis is still under debate. During the prognosis study, AML patients with CEPBA mutations showed selective upregulation of some miRNAs, including several members in the miR‐181 family, compared to patients without mutation.[[qv: 7d]] In our previous work, we had found target sequences of C/EBPα‐p30 in the promoter upstream of the miR‐181a‐1 gene, and subsequently confirmed its transactivity by C/EBPα‐p30 via luciferase array.[[qv: 7c]] Since miR‐181a is known to be tumor suppressive, such a discovery provided possible explanation of the clinical observations.

The upregulation of miR‐181a can occur via different routes, of which the most direct one is the transfection of plasmids carrying miR‐181a gene sequence. As demonstrated in Figure S2 (Supporting Information), compared to the direct approach, the upregulation of miR‐181a by C/EBPα‐p30 involves several additional steps, including a full round of protein expression and relocation into nucleus. With a longer intracellular pathway, the indirect approach by the promotion of endogenous miR‐181a expression would require more cellular activities for an equivalent upregulation than the direct delivery of exogenous gene, potentially burdening the cell. Yet, once transcribed or translated, the CEBPA mRNA and C/EBPα dimer could continue to function and eventually generate more than one copy of protein or miR‐181a. The cascade of their activities provides a leverage effect, which may result in more efficient upregulation per expression of plasmid than the direct route, i.e., miR‐181a vector, of which each transcription generates only one copy of miR‐181a. To compare the efficiency between direct and detoured upregulation routes, we constructed several pIRES‐EGFP plasmids carrying miR181a gene and wild‐type/mutated CEBPA genes, respectively. The plasmids were delivered into an AML cell line (KG‐1a) and the expression of miR‐181a was measured 24 h later. KG‐1a cells were selected because they do not transcribe the endogenous CEBPA mRNA,^8^ minimizing the interference from endogenous expression. For transfection, both conventional bulk electroporation using a commercial system and NEP have been carried out. Despite the wide use and success in adhesive cell lines with small plasmids, the conventional bulk electroporation resulted in severe cell death in our case (leukemia cell with larger plasmid) and could not induce significant expression of plasmids in repeated attempts (see Figure S3–S6, Supporting Information for more details). Similar observations have also been made by other groups.^14^ On the other hand, NEP transfection readily showed high fluorescence imaging of the reporter protein in individual cells after transfection of pIRES‐EGFP plasmid carrying N‐mut CEBPA as shown in Figure S6 (Supporting Information). Compared to conventional bulk electroporation (Figure S5, Supporting Information), all three NEP‐transfected cells examined showed fluorescence from the reporter gene, proving the efficiency of NEP delivery.

The reporting EGFP gene on pIRES plasmid shares its promoter with the cloned gene, therefore allowing GFP fluorescence to serve not only as a qualitative marker for plasmid expression, but also a quantification index of gene transcription activity from the plasmids. To detect the intracellular miR‐181a level in transfected cells, miR‐181a MB with Cy3 as a reporter was designed and used for the follow‐up NEP. During fluorescence micro­scopy, signals of both EGFP and Cy3 channels from transfected cells were recorded, and plotted according to transfected gene type in **Figure** **3**. Linear regression with least square estimator was used to calculate the relationship between the two signals as an estimation of the upregulation efficiency by different plasmids. The results of regressions are also shown in Figure 3.

**Figure 3:**
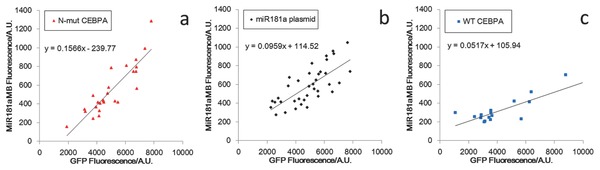
Relationship between miR‐181a level and vector expression for different genes: a) N‐terminal CEBPA mutant for C/EBPα‐p30 pathway; b) miR‐181a gene for direct upregulation; c) wild‐type CEBPA gene as a positive control.

The slopes of linear regression between miR‐181a signal and EGFP signal from plasmids are 0.1566 for N‐mut CEBPA gene, 0.0959 for miR‐181a gene, and 0.0517 for wild type CEBPA gene. The differences of the slopes indicate the efficacy of plasmids and imply that the indirect upregulation route by C/EBPα‐p30 is more efficient than the direct upregulation route of miR‐181a. The wild‐type CEBPA gene served as a positive control as it translated more p42 isoform than p30, therefore only shows a minor upregulation effect compared to the N‐terminal frame shift mutant.

A new single‐cell analysis platform based on NEP‐MB‐Optical Tweezers was designed and demonstrated. The system integrates the precise delivery ability of NEP and intracellular RNA analysis ability of MBs. The platform could provide quantitative analysis of multiple mRNAs/miRNAs in a single cell without cell lysis or fixation, offering greater flexibility than PCR and immunocytochemistry‐based methods. In addition, the platform also offers delivery of precise dosage of genes or other types of stimulus into individual cells before MB‐based living cell interrogation. To demonstrate its application, the downregulation of DNMT3A/B mRNAs by miR‐29b in Kasumi‐1 cells was verified at single‐cell level on the platform, showing multi‐targeting ability in individual cells. The platform has also been used to analyze and compare the upregulation efficiencies of miR‐181a through different routes, showing an indirect approach could produce a stronger upregulating effect. This single‐cell analysis platform can handle a cell population from single to ≈10^2^ cells, and achieve high transfection efficiency for large genes when conventional methods fail. The analysis does not require cell fixation or lysis, which allows further interrogation of the targeted living cells. These unique features would be very important in the case of rare cell analysis (such as circulating tumor cells and stem cells).

## Supporting information

As a service to our authors and readers, this journal provides supporting information supplied by the authors. Such materials are peer reviewed and may be re‐organized for online delivery, but are not copy‐edited or typeset. Technical support issues arising from supporting information (other than missing files) should be addressed to the authors.

Supplementary
