# Supplementary material for: Nanochannel Electroporation as a Platform for Living Cell Interrogation in Acute Myeloid Leukemia
Source: Adv Sci (Weinh). 2015 Jul 16;2(12):1500111. doi: 10.1002/advs.201500111 (PMC5115302; doi:10.1002/advs.201500111)
Supplement: Supplementary file 1 — Supplementary [file ADVS-2-0o-s001.pdf]

Copyright WILEY-VCH Verlag GmbH & Co. KGaA, 69469 Weinheim, Germany, 2015.

ADVANCED SCIENCE
Open Access

Supporting Information

for *Adv. Sci.*, DOI: 10.1002/advs.201500111

Nanochannel Electroporation as a Platform for Living Cell Interrogation in Acute Myeloid Leukemia

*Xi Zhao, Xiaomeng Huang, Xinmei Wang, Yun Wu, Ann-Kathrin Eisfeld, Sebastian Schwind, Daniel Gallego-Perez, Pouyan E. Boukany, Guido I. Marcucci, and Ly James Lee**

Supporting Information

Nanochannel Electroporation as a Platform for Living Cell Interrogation in Acute Myeloid Leukemia

Xi Zhao, Xiaomeng Huang, Xinmei Wang, Yun Wu, Ann-Kathrin Eisfeld, Sebastian Schwind, Daniel Gallego-Perez, Pouyan E. Boukany, Guido I. Marcucci[#] and L. James Lee[*]

Dr. X. Zhao, Dr. X. Huang, Dr. X. Wang, Dr. Y. Wu, Dr. D. G. Perez, Dr. P. E. Boukany, Prof. L. J. Lee

Center for Affordable Nanoengineering of Polymeric Biomedical Devices, The Ohio State University, Columbus, Ohio, 43212, USA

Email: lee.31@osu.edu

Dr. X. Zhao, Prof. L. J. Lee

William G. Lowrie Department of Chemical and Biomolecular Engineering, The Ohio State University, Columbus, Ohio, 43210, USA

Dr. A-K Eisfeld, Dr. S. Schwind, Dr. G. I. Marcucci

Department of Internal Medicine, Comprehensive Cancer Center, The Ohio State University, Columbus, Ohio, 43210

Operation of NEP

A sealed and sterilized NEP device was filled with 50 µL of sterilized phosphate buffered saline (PBS) in each reservoir and then exposed to a low degree of vacuum to assist the wetting of micro/nanochannels. The vacuum-treated device was examined under a microscope to make sure all microchannels were filled. The PBS in the cell side reservoirs was then substituted with RPMI-1640 medium with FBS by three washes.

For miRNA and MB transfection, a concentrated solution of either miR-29b mimic, mixture of DNMT3A/B MBs, or miR181a MB was added to the nucleic acid side reservoir, and then diluted by PBS to a final concentration of 1 µM. The device was incubated at 37ºC for 20 minutes to allow the diffusion of miRNA or MBs into microchannels as well as to warm up the fluid in the cell side reservoir. For plasmid transfection, a concentrated solution of plasmid was added into the nucleic acid reservoir at a final concentration of 0.5 µg/µL. Solution in the reservoir was thoroughly mixed by pipetting and a diffusion time of at least 20 minutes was allowed before poration. After incubation, 10 µL of cell suspension (Kasumi-1 or KG-1a) at a density of 10^5 cells/mL was added to the cell side reservoir. The final cell density on device was about 5×10^3 cells/mL to avoid crowding of cells on the bottom of device.

The loaded device was mounted onto an inverted fluorescence microscope (Olympus IX-81, with a 60× oil immersion objective lens of NA 1.42) and optical tweezers were used to capture individual cells in the reservoir and relocate them into individual microchannels against the tips of nanochannels.[1] The typical maneuver time of each cell ranged from 1 to 2 minutes, depending on the initial locations of cells. A pair of platinum wires connected to an electroporation power supply (Gene Pulser Xcell, Bio-Rad) was immersed into the two

reservoirs as electrodes. Two to five electric pulses between 220-250V, each lasting 5 or 10 miliseconds, were delivered with the square wave protocol to conduct cell transfection. When multiple nanochannel connections exits between two microchannels (see **Figure 1**), the electrophoresis was dominated by the path of lowest resistance, therefore no adjustment on the pulsing condition was needed as long as the cell was only touching one nanochannel or the other nanochannels are significantly longer than the touching nanochannel.

After transfection, the nucleic acid reservoir was flushed with PBS for at least 3 times to remove undelivered material. In the case of miR-29b or plasmid transfection, the device was placed in a 100 mm petri dish and incubated overnight at 37ºC and 5% CO_2 before NEP delivery of MBs. After delivery of MBs, the device was cultured for 40 minutes before fluorescence imaging on a Nikon Ti-E inverted fluorescence microscope with an EMCCD camera (Photometric Evolve 512) and 100× NA1.40 oil immersion objective lens. In each case, MB fluorescence was collected with the filter for its corresponding dye: GFPHQ filter cube for FAM; Cy5 filter cube for Cy5; TxRed filter cube for Cy3.

Structure of CEBPA

CEBPA gene encodes an intronless transcription factor that plays vital function in hematopoiesis. The mRNA of CEBPA contains two initiation codons and can be translated into two polypeptides. The longer one (C/EBPα-p42) weights 42kDa and is the dominating isoform. Although only translated in smaller quantity, the shorter, 30kDa isoform is also naturally translated [2]. The full-length p42 peptide consists of multiple trans-activation zones [3], a DNA binding domain (DBD) and a leucine zipper (L-ZIP) motif. The p30 isoform loses the first 119

amino acid residuals (including TAD1 trans-activation zone) and is previously considered to be functionless [2a, 3].

Conventional electroporation and PCR

Delivery of CEBPA plasmids with (pIRES-N mut CEBPA-EGFP) and without GFP reporter (pMSCV empty vector, pMSCV-C mut CEBPA, pMSCV-N mut CEBPA and pMSCV-WT CEBPA) into KG-1a cells were carried out with a Neon® transfection system (Life Technologies) following recommended protocols. A single 20ms pulse at 1650V was applied and post-transfected cells were deposited into 6-well plates before viability and expression check 24 hours later. Electroporation in the absence of plasmid was also conducted as a negative control.

Real time qRT-PCR was used to check the expression level of miR-181a in untreated and electroporated cells. One day after electroporation, cell lysis and total RNA collection was conducted with TRIzol® reagent (invitrogen). MiR-181a was reverse-transcripted into cDNA and subsequently measured by PCR with RNU48 as internal control by TaqMan® assay on an Applied Biosystems StepOnePlus™ Real-Time PCR System

Vector expression and cell viability

Viability of conventionally electroporated KG-1a cells were examined 24 hours after transfection by two methods: Trypan blue exclusion and MTS assay (Promega). For Trypan blue staining, 200μL (10% of culture medium volume) of 0.4% Trypan blue solution in PBS was added into each well of the electroporated cell culture on the 6-well plate and gently mixed by pipetting. 10μL of culture suspension was immediately loaded onto a hemacytometer and

visually checked under a microscope. The total and stained cells were counted and the viability was calculated by dividing the non-stained cell number with the total cell count. For MTS assay, the culture medium containing electroporated cells was dispensed on a 96-well plate at a volume of 100μL per well and 20μL of MTS solution was added per well. The plate was cultured at 37ºC for 2 hours and absorbance measurements at 492nm were taken on a microplate reader (Tecan GENios Pro). Absorbance at 640nm was also measured to allow subtraction of background, and the viability of cells was normalized to an untreated control group.

Viability of individual cells 1 day after NEP was checked with calcein AM/EthD-1 staining (Invitrogen): A working solution of 2μM calcein AM and 4μM EthD-1 was added to the reservoir and the device was cultured at 37ºC for 30 minutes before fluorescence check. The process has been described in detail previously [4].

For the verification of transfection, the N-mut CEBPA-EGFP plasmid was used for both conventional electroporation and NEP. Nucleus staining with DAPI was performed 24 hours after poration and cells were fixed with 4% paraformaldehyde solution before differential interference contrast (DIC) and fluorescence imaging on a laser scanning confocal microscope (Olympus Flowview 1000).

Cell line, plasmids and miRNAs

Each miRNA can have multiple targets and thus involves in many pathways. For example, one of mechanisms underlying tumor-suppressive effect of miR-29b is that it can induce hypomethylation of DNA by directly targeting DNA methyltransferases (DNMTs) A and B, which we confirmed in this work. In addition, miRs are under the precise regulation by

transcriptional factors. We have previously reported that miR-181a promoter contains CEBPα binding site.[5]

Acute myeloid leukemia (AML) cell lines, Kasumi-1 and KG-1a, were obtained from the American Type Culture Collection (ATCC; Manassas, VA) and used in this study. Kasumi-1 cells were cultured in RPMI-1640 medium (Life technologies) with 20% fetal bovine serum (FBS, Sigma-Aldrich), while KG-1a were cultured in the same medium with 10% FBS. Cell density of each cell lines was maintained between 2×10^5 and 2×10^6 cells/mL.

Expression vectors (pMSCV & pIRES-EGFP, Clontech Laboratories. LLC) containing wild type and mutated CEBPA genes (H24Afs for N-terminal mutation and R300L for C-terminal mutation[5]) were provided by collaborators from the Ohio State University Medical School. A pIRES-miR-181a-EGFP vector was also constructed for semi-quantitative comparison of the upregulation effect on miR-181a expression. Synthetic human miR-29b mimic (item ID PM10103) was purchased from Ambion (Life Technology, NY) and stored at -70ºC until use.

Design of Molecular Beacons

Several molecular beacons were used in this study. Molecular beacons that target DNA (cytosine-5-)-methyltransferase 3A/B (DNMT3A/B) mRNAs were purchased from TIB MOLBIOL, LLC and have the following designs:

DNMT3A:

6FAM-CGCGATCCGCGATTGCACCATAAGATGTCCTCTTGATCGCGGATCGCG-BHQ1

DNMT3B:

Cy5-CGCGATCCGCGATTTCACGGTTCCAACAGCAATGATCGCGGATCGCG--BBQ

The two MBs were constructed with different dye/quencher pairs to allow simultaneous detection of both in one cell with minimal fluorescence cross-talk.

A MB for microRNA with Locked Nucleic Acid (LNA) structure was purchased from Sigma-Aldrich with the sequence listed below and capital letters representing the LNA unit.

miR-181a MB: 5'-Cy3-accgcg-ActCacCgaCagCgtTgaAtgtt-cgcggt-BHQ2-3'

Plasmid transfection by conventional electroporation

Despite their wide range of applications in transient gene transfection for various adherent cell lines, conventional electroporation was not able to reliably deliver plasmids constructed in this study into KG-1a cells. Severe loss of cell viabilities 24 hours after electroporation attempts (with pMSCV vectors) were observed when transfected with commercial electroporation following recommended protocol. The trend was confirmed in independent experiments with different viability measurement protocol (Trypan blue exclusion and MTS assay). Electroporation without plasmid and untreated cells were also tested as positive and negative control. **Figure S3** summarizes the results. Both methods concluded that the viabilities of cells after electroporation with plasmids dropped significantly, to below 60%, suggesting severe damage was made to a significant portion of electroporated cells.

To examine whether the live cells after conventional electroporation expressed the genes carried on the plasmids, a pIRES-N mut CEBPA-EGFP vector was used in a separate electroporation and fluorescence from GFP reporter in the remaining cells was checked on a laser confocal microscope after staining the nucleus with DAPI dye. Fluorescence and DIC images from several cells are available in **Figure S5**, with all cells exhibiting minimal GFP signal.

From our experience, plasmid transfection by conventional electroporation often does not work very well with leukemia/lymphoma cell lines, and the efficiency drops significantly as the size of the plasmid increases. Similar observations have been made by other groups. For example, Bockstaele et al. also reported around 50% survival and less than 20% overall transfection efficiency by nucleofecting with 2ug pmax-GFP with 6 different programs in CLL cells (see their supplementary figure 1)[6]. In fact, in 2000, Shimokawa et al reported that introduction of DNA into a promonocytic cell line U937 by electroporation can cause massive cell death[7].

The low transfection and expression efficiency combined with the low cell viability after electroporation compromised the tool's ability on examining the potential linkage between CEBPA point mutation and miR-181a expression level in KG-1a cells. With the majority of cells either undergoing apoptosis or not restoring CEBPA expression, the miR-181a level in post-transfected cell populations fluctuated and showed greater variation between transfection batches than between different plasmids (see **Figure S4**). Therefore, the exploration on the effects of CEBPA mutant by conventional electroporation is inconclusive. An alternative transient transfection method needs to be used for such purpose.

References

[1] P. E. Boukany, A. Morss, W.-C. Liao, B. Henslee, H. Jung, X. Zhang, B. Yu, X. Wang, Y. Wu, L. Li, K. Gao, X. Hu, X. Zhao, HemmingerO, W. Lu, G. P. Lafyatis, L. J. Lee, *Nature Nanotech.* **2011,** *6*, 747.
[2] a) F. T. Lin, O. A. MacDougald, A. M. Diehl, M. D. Lane, *Proceedings of the National Academy of Sciences* **1993,** *90*, 9606; b) V. Ossipow, P. Descombes, U. Schibler, *Proceedings of the National Academy of Sciences* **1993,** *90*, 8219.
[3] C. Nerlov, E. B. Ziff, *Genes & Development* **1994,** *8*, 350DOI 10.1101/gad.8.3.350.
[4] X. Zhao, Y. Wu, D. Gallego-Perez, K. J. Kwak, C. Gupta, X. Ouyang, L. J. Lee, *Analytical Chemistry* **2015**, DOI 10.1021/ac503366w.

[5] C. J. Hickey, S. Schwind, H. S. Radomska, A. M. Dorrance, R. Santhanam, A. Mishra, Y. Z. Wu, H. Alachkar, K. Maharry, D. Nicolet, K. Mrozek, A. Walker, A. M. Eiring, S. P. Whitman, H. Becker, D. Perrotti, L. C. Wu, X. Zhao, T. A. Fehniger, R. Vij, J. C. Byrd, W. Blum, L. J. Lee, M. A. Caligiuri, C. D. Bloomfield, R. Garzon, G. Marcucci, *Blood* **2012,** *121*, 159.
[6] F. Van Bockstaele, V. Pede, E. Naessens, S. Van Coppernolle, V. Van Tendeloo, B. Verhasselt, J. Philippé, *Leukemia* **2008,** *22*, 323.
[7] T. Shimokawa, K. Okumura, C. Ra, *Biochemical and Biophysical Research Communications* **2000,** *270*, 94.

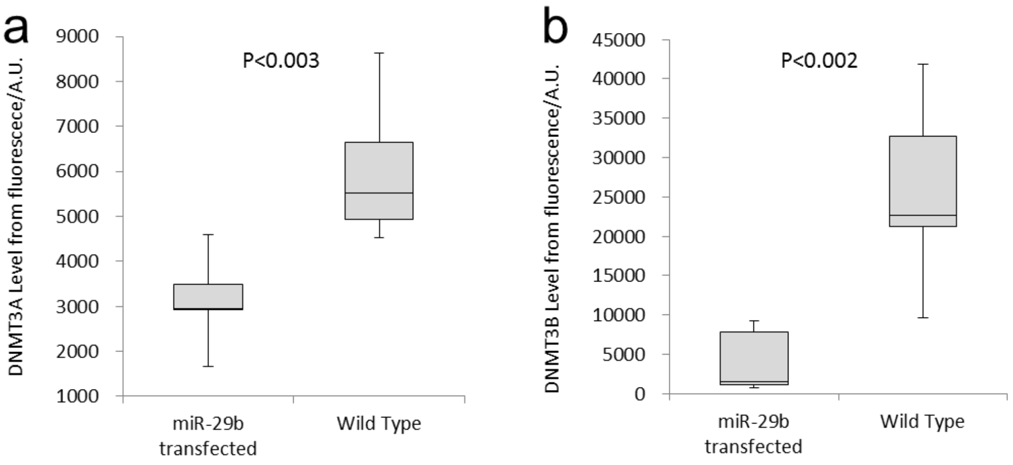

Figure S1. Box plot of a) DNMT3A and b) DNMT3B mRNA level in Kasumi-1 cells 24 hours after transfection of miR-29b showed downregulation of both mRNAs (n=7 for normal cell, n=5 for transfected cells).

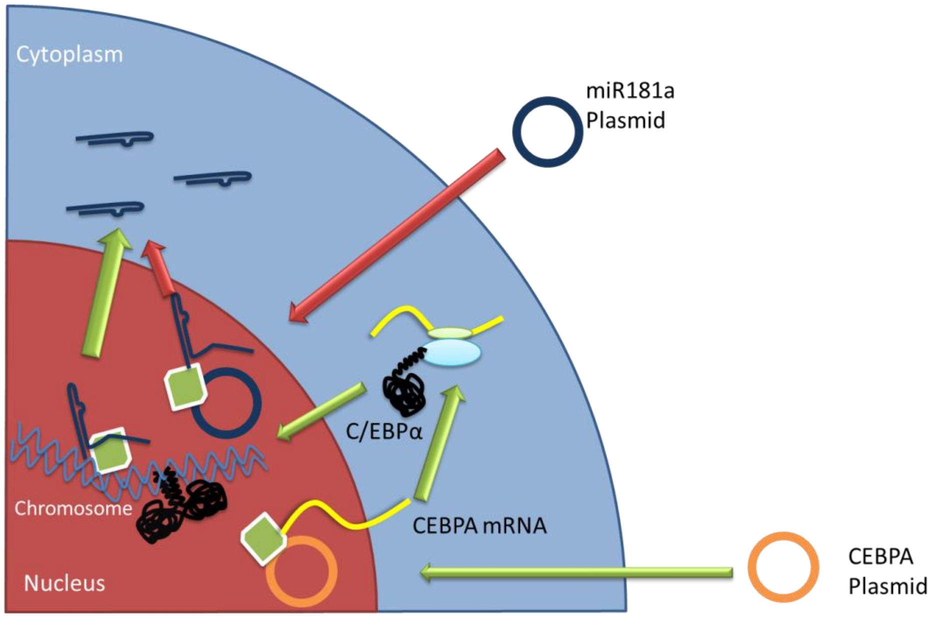

Figure S2. Pathway of miR-181a upregulation by CEBPA plasmid (in green arrow) and miR-181a plasmid (in red arrow).

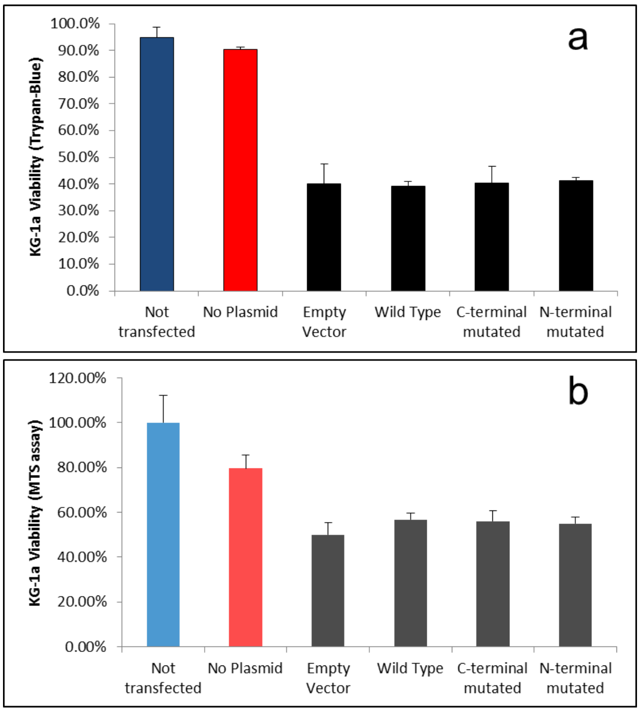

Figure S3. KG-1a Cell viabilities 24 hours after electroporation by Neon®. Measured by: a) Trypan Blue and manual count on hemacytometer; b) MTS assay on plate reader. No Plasmid represents electroporation following normal procedure, but without adding plasmid.

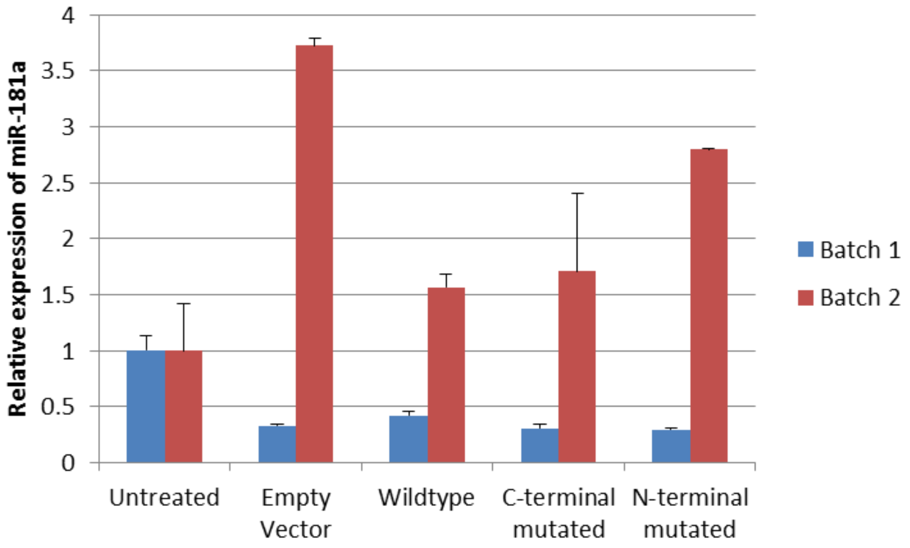

Figure S4. Expression level of miR-181a from different batches of transfected KG-1a cells with pMSCV vectors measured by qRT-PCR. Expression levels were converted from ΔΔCt values with RNU48 as internal control and 2 as the base.

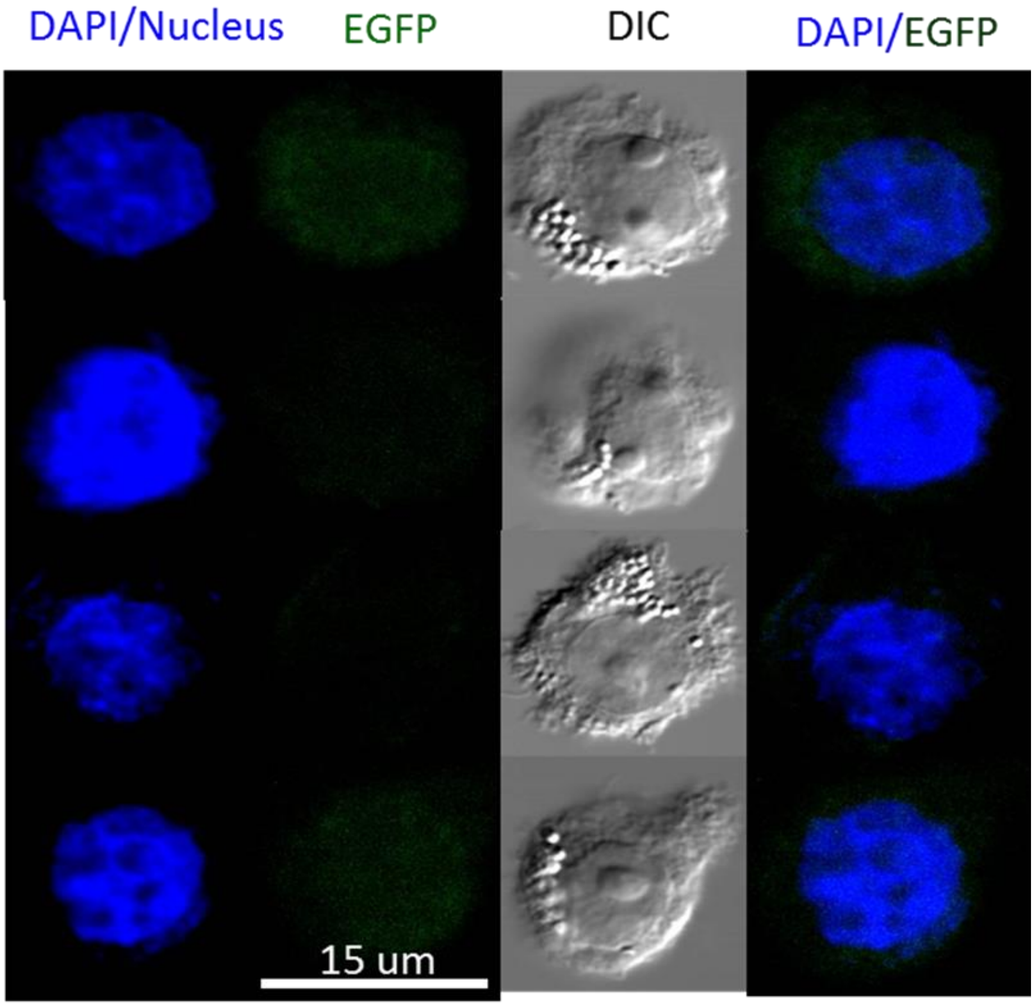

Figure S5. Confocal fluorescence and DIC images of selected KG-1a cells 24 hours after conventional electroporation of pIRES-N mut CEBPA-EGFP plasmids. The absence of EGFP expressions in all 3 cells shown here indicated that the transfection efficiency by conventional electroporation in this case was very low.

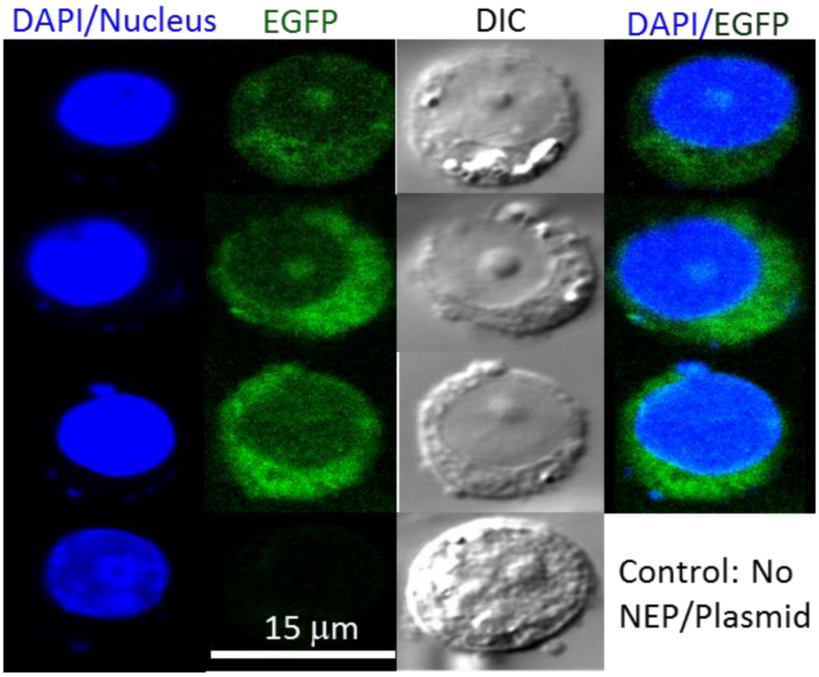

Figure S6. Confocal fluorescence and DIC images of selected KG-1a cells 24 hours after NEP of pIRES-N mut CEBPA-EGFP plasmids. Contrary to the observation after conventional electroporation, all 3 cells transfected by NEP showed EGFP expression, indicating that the efficiency of NEP was high. The control cell was not transfected and showed no green fluorescence signal.
